# Analysis of the Clinical Course of Primary Sclerosing Cholangitis in Paediatric Population—Single Center Study

**DOI:** 10.3390/medicina57070663

**Published:** 2021-06-27

**Authors:** Sabina Wiecek, Alicja Wojtyniak, Barbara Pindur, Magdalena Machnikowska-Sokołowska, Katarzyna Gruszczyńska, Urszula Grzybowska-Chlebowczyk

**Affiliations:** 1 Department of Paediatrics, Faculty of Medical Sciences, Medical University of Silesia, 16 Medykow Street, 40-752 Katowice, Poland; urszulachlebowczyk@wp.pl; 2 Student Scientific Club, Department of Paediatrics, Faculty of Medical Sciences, Medical University of Silesia, 40-752 Katowice, Poland; dopracyinauki@gmail.com (A.W.); barbara.d.pindur@gmail.com (B.P.); 3 Department of Radiology and Nuclear Medicine, Faculty of Medical Sciences, Medical University of Silesia, 40-752 Katowice, Poland; magdams@onet.pl (M.M.-S.); kgruszczynska@poczta.onet.pl (K.G.)

**Keywords:** primary sclerosing cholangitis, IBD, children, clinical course

## Abstract

*Background and Objectives:* Primary sclerosing cholangitis (PSC) is a rare cholestatic disease of the liver of unknown etiology, severe course and poor prognosis. PSC most often co-occurs with inflammatory bowel diseases (IBD), especially with ulcerative colitis (UC). The aim of the study was the analysis of the clinical course of primary sclerosing cholangitis in children, hospitalized in the Gastroenterology Unit in Katowice. *Materials and Methods:* The analysis included 30 patients, aged from 7 to 18 years, 21/30 boys (70%) and 9/30 girls (30%), diagnosed with PSC in the years 2009–2019. The analysis included the age at diagnosis, clinical symptoms, course of the disease, coexisting diseases, laboratory and imaging results, and complications. *Results:* The average age at diagnosis was 13 years. 22/30 (73.3%) patients suffered from UC, 4/30 (13.3%) were diagnosed with Crohn’s disease (CD), 2/30 (6.66%) with Eosinophilic Colitis (EC). 2/30 patients (6.66%) had no clinical evidence of coexistent IBD to date. In addition, 7/30 (23.3%) had an overlap syndrome of primary sclerosing cholangitis/autoimmune hepatitis. When PSC was detected before IBD (6/30–20%), patients had complications more often compared to those diagnosed with IBD first or PSC and IBD at the same time. At the moment of diagnosis 6/30 (20%) patients presented with abdominal pain, which was the most common symptom, 3/30 (10%) jaundice, while 17/30 (56.6%) were asymptomatic but had abnormal results of the laboratory tests. *Conclusions:* Monitoring liver markers in IBD patients is important since most PSC cases are asymptomatic and their elevation might be the first sign of the disease. Patients diagnosed with PSC before IBD diagnosis are more likely to have a more aggressive course of the disease.

## 1. Background

Primary sclerosing cholangitis (PSC) is a chronic cholestatic liver disease of complex etiology which leads to the damage of intra- and extrahepatic bile ducts. The etiopathogenesis is characterised by the following factors: genetic (HLAA1, B8 and DR3 genotypes), autoimmune, infectious, and environmental (the impact of diet and the gastrointestinal microbiome). The greatest morbidity rate is observed in Europe and North America, and accounts for 0.5–1.0/100,000/per year. Even though the highest morbidity rate concerns people aged between 20 and 40, PSC has been more frequently diagnosed in paediatric patients. (0.1–0.5/100,000/annum) [1,2,3,4,5,6]. The clinical picture is not characteristic. 40–60% of patients are asymptomatic, and it is the observed abnormal parameters of cholestasis and damage to the liver that potentially indicate primary sclerosing cholangitis. Some of the patients present with jaundice of the skin and the sclera, pruritus, weakness, loss of body mass, fatigue, pain in the right epigastrium and/or episodes of fever. PSC is a progressive disease, leading to cirrhosis and hepatic insufficiency. 50% of patients with PSC require liver transplant within 10–15 years of diagnosis. 50–90% of patients with PSC have co-existing Inflammatory Bowel Disease (more often ulcerative colitis than Crohn’s disease). The lesions more often affect the right large intestinum, with unaffected rectum. On the other hand, only 5–10% of patients with ulcerative colitis and 2–5% of those with Crohn’s disease have co-existing PSC. Patients with PSC often have autoimmune comorbidities, such as type 1 diabetes, coeliac disease, autoimmune pancreatitis/thyroiditis, glomerulonephritis and/or arthritis. Autoimmune hepatitis co-exists in 25–35% of patients with PSC [7,8,9,10,11,12]. The results of the laboratory tests reveal elevated parameters of cholestasis and liver damage. 40–50% of patients have increased levels of IgM and IgG, and 20–50% present with anti-nuclear antibodies and/or smooth muscle antibodies [13,14,15,16]. The ultrasound of the liver and bile ducts may help with the final diagnosis; however, it is often negative. In the magnetic resonance cholangiopancreatography (MRCP) the characteristic picture of constrictions with subsequent expansions of the intra- and extrahepatic bile ducts can be seen. The endoscopic retrograde cholangiopancreatography (ERCP) is performed when there is clinical uncertainty, treatment is necessary (sphincterotomy or stenting) and/or there is suspicion of cholangiocarcinoma, and therefore cytology is required. Similarly, liver biopsy with histopathology is only performed in the case of diagnostic uncertainty, when autoimmune hepatitis and/or small duct PSC is suspected. Histopathology shows fibrosis around bile ducts—onion-skinning” in 20–40%, portal inflammatory infiltrate, proliferation of bile ducts, and consequently ductopenia [17,18,19,20,21,22,23,24]. Primary sclerosing cholangitis is linked to a higher risk of developing colorectal cancer (9% after 10 years of diagnosis, 31% after 20 years and 50% after 25 years of diagnosis), cholangiocarcinoma and/or gallbladder cancer (400 times higher than the population risk). Close to 50% of gallbladder polyps in patients with PSC is malignant. 1/3 of cholangiocarcinoma in adults is diagnosed at the same time as the diagnosis of PSC [25,26,27,28,29,30,31]. The treatment of primary sclerosing cholangitis involves ursodeoxycholic acid, which reduces the risk of dysplasia within the large intestine; however, its use is controversial. The efficacy of vancomycin in the treatment of patients with PSC has not been successfully proven. ERCP is a procedure used to treat biliary stenosis and gallstones. Liver transplant is performed in the case of recurrent cholangitis, end-stage liver failure and/or treatment-resistant pruritus. 15–45% of paediatric patients with diagnosed PSC require liver transplant in the first 12 months of diagnosis [32,33,34].

The aim of this study was the analysis of the clinical course of primary sclerosing cholangitis in children hospitalized in the Gastroenterology Unit, Department of Paediatrics, Medical University of Silesia in Katowice.

## 2. Methods

We retrospectively reviewed the medical records on all known PSC patients with disease onset prior to 18 years of age treated in 2009–2019 in the Gastroenterology Unit, Department of Paediatrics, Medical University of Silesia in Katowice. The analysis included 30 patients, aged from 7–18 years (mean age 13.8 years), 21/30 boys (70%) and 9/30 girls (30%) with diagnosed PSC. PSC was diagnosed based on the clinical presentation, results of the laboratory tests and MRCP. The assessment included the clinical symptoms and signs at the PSC diagnosis, the presence of associated IBD, the presence of AIH and any other coexisting autoimmune diseases as well as other non-associated diseases diagnosed at any time during the available follow-up; laboratory and imaging (USG and MRCP) examinations, liver histopathological examination, the occurrence of complications and the applied treatment. As for the IBD data, we included the type of IBD (ulcerative colitis, Crohn’s disease, Eosinophilic Colitis), the age at IBD diagnosis and the location of the disease.

Inflammatory bowel diseases were diagnosed based on the clinical presentation and the results of the laboratory, endoscopic and histopathological tests as per the Porto criteria of 2005, with the modifications of 2014 and including the Paris classification [35].

Eosinophilic colitis was diagnosed based on the clinical presentation and the results of the colonoscopy and histopathology of the biopsies, assessed using the Whittington scale [36].

Other comorbid diseases in the patients and their family members were recorded when available in medical records.

Laboratory results were collected from the moment of diagnosis and after a median follow-up of three years. The analysis included alanine aminotransferase (ALT), aspartate aminotransferase (AST) activities, total serum bilirubin (BLB) concentration, gamma glutamyltranspeptidase (GGT) activity and AST-to-platelet-ratio Index (APRI).

All children underwent ultrasonography examination as a first line diagnostic modality, (Samsung RS85, and Loqic S7 with covex 3–10 mHz transducer), which was used for general liver, portal system, pancreas, gallbladder and the bile ducts checks. US was also used for the bowel examination in children with IBD. The thickening of bowel walls (especially in the ileo-colic junction), increased vascularization, enlargement of the regional lymph nodes and signs of inflammatory reactions in adjacent soft tissues were taken into consideration.

As for the imaging symptoms potentially indicating PSC, we considered the widening of the common bile duct or/and the common hepatic duct, strictures of bile ducts, irregular “beaded’ outline of the bile ducts, as well as thickening of their walls.

MRCP was performed on 1,5T (OPTIMA MR 450w GE Healthcare), a phased-array body coil was used with the following protocol: localizers—Free-breathing 3-plane Loc with auto tracker prescription, coronal FIESTA SSFSE BH; axial T2 & T2 FS; 3D coronal navigated MRCP without contrast administration, with MIP reformation MRCP parameters: TR, ms: 3000–5000, TE, ms: 520–766, FOV, cm: 30–40 depending on child size, Slice thickness, mm: 1,4, Scan time, min: 4:00–4:30. In children below 7–8 years of age sedation was needed. The images obtained were reviewed on the dedicated workstation adw4.6 by paediatric radiologists with at least 10 years’ experience.

The statistical analysis included descriptive methods and standard tests of association. Percentages and means (or medians in non-normally distributed data) were used to describe the clinical data. The tests of association included The Wilcoxon Signed-Ranks Test for laboratory results and chi-square or Fisher exact tests for categorical data. Level of statistical significance was set on *p*-value of 0.05. Statistical analyses were completed using the Statistica 13 software.

## 3. Results

The examined group included 21/30 (70%) boys and 9/30 (30%) girls. The mean age of PSC diagnosis was 13 years. In 5/30 (16.6%) of the children, primary sclerosing cholangitis was observed in the patients below the age of 10.

Only 13/30 (43%) patients presented with signs or symptoms at the moment of diagnosis, including abdominal pain, weight loss, jaundice, malaise and raised temperature. Abdominal pain was the most commonly observed symptom, in 6/30 (20%). The first onset of hepatic lesions, preceding the diagnosis of inflammatory bowel disease was reported in 6/30 (20%) of the patients (PSC 5/6, overlap syndrome PSC/AIH 1/6).

IBD was diagnosed in 28/30 (93.3%) of cases (ulcerative colitis in 22/30–73%, Crohn’s disease in 4/30–13.3%, eosinophilic colitis in 2/30–6.66%) with a mean age of diagnosis of 12 years. 2/30 patients (6.66%) had no clinical evidence of coexistent inflammatory bowel disease to date. As per the Paris classification, the E4 location—lesions within the whole of the large intestine was predominant in patients with ulcerative colitis (16/22–72.7%), compared with 2/4 (50%) A1L3B2 of patients with Crohn’s disease. In 3/30 (10%) patients with diagnosed inflammatory bowel disease, no lesions within the rectum were observed—1 patient with UC, 1 with CD, 1 with EC. Specific results considering the parts of the colon affected by IBD are depicted in Table 1.

The overlap syndrome of PSC/AIH occurred in 7/30 (23%) patients. 1/30(3.33%) patients with primary sclerosing cholangitis suffered from autoimmune pancreatitis. No other autoimmune disease was reported.

Gastritis and/or duodenitis was mentioned in 10/30 (33%) of the cases. 4/30 (13.3%) patients have diagnosed lactose intolerance and 3/30 (10%) patients have diagnosed food allergies. 1/30 (3.33%) patients with PSC/AIH/IBD was diagnosed with osteoporosis, probably related to underlying diseases and the applied treatment.

Elevated activities of GTP were observed in all the patients with diagnosed PSC (mean activity at 73 U/l). Increased activity of alanine aminotransferase was observed in 21/30 (70%) patients and of asparagine aminotransferase in 23/30 (76.6%). Abnormal values of the APRI index were reported in 6/30 patients (16.6%), all of whom had advanced liver conditions such as cirrhosis/portal hypertension.

In the follow-up, we observed a decrease in mean GGT activities from 73 U/l to 37 U/l (*p* < 0.05). ALT, AST activities, BLB concentration and APRI index results did not change significantly. The detailed results of laboratory tests are depicted in Table 2.

Abnormal values of ANCA antibodies were observed in 8/28 (28.5%) patients and ASCA-positive antibodies in 4/28 patients, 3 of whom had Crohn’s disease and 1 ulcerative colitis.

MRCP was performed on all 30 patients at diagnosis and the characteristic features of PSC were observed in all of them, which included multifocal narrowing or strictures and dilatations of intra and/or extrahepatic bile ducts, with beading or string of pearl appearance. The follow-up tests were conducted at 6-month intervals. Abdominal USG was conducted in all patients but only 9/30 (32%) US showed various bile duct abnormalities. Liver biopsy was performed on 13/30 (43.8%) patients with suspected AIH. In 7/13 (54%) bioptates of liver- histopathological examinations revealed changes typical of either PSC/AIH. In 5/13 (38.5%) biopsies there were no characteristic changes for AIH/PSC and 1/13 (7.7%) biopsy was non-diagnostic.

The complications were observed in 8/30 (27%) patients and involved the occurrence of portal hypertension 2/30 (6.6%), liver cirrhosis 2/30 (6.6%) and biliary complications defined as a critical stricture of bile ducts which required biliary prothesis through ERCP 5/30 (16.6%). We did not register any cholangiocarcinoma or death in the group of PSC patients. However, elevated levels of Ca19-9 tumour marker were observed in 2 patients. 1 of them required a liver transplant at 19 years due to the progressing PSC, recurrent acute cholangitis and liver failure. As for the second patient, no atypical cells within the bile ducts were observed in the cytological and histopathological tests and he remains under close gastro-and oncological care.

When PSC was detected before IBD (6/30–20%), patients had complications more often compared to those diagnosed with IBD first or at the same time (*p* = 0.007). Moreover, the complications were observed more often in children with changes characteristic of PSC visible in their first US examination than in patients without the mentioned abnormalities in the first US (*p* = 0.005). We didn’t find statistical significance correlation between occurrence of liver’s complication and Inflammatory Bowel Disease activity and extent of changes in large intestine Table 3.

All PSC patients were treated with ursodeoksycholic acid (UDCA). 27/30 (90%) children received 5-aminosalicylic acid (5-ASA) as IBD treatment. 11/30 (37%) patients took azathioprine (AZA) for either AIH or UC. Infliximab (monoclonal antibody biologic drug) was prescribed for 1/30 (3.3%) patient with severe UC.

## 4. Discussion

Pediatric primary sclerosing cholangitis has a chronic, progressive and severe course. Nearly half of the patients will develop an adverse liver condition within 10 years of diagnosis. Compared to the descriptions of adult-onset PSC, the pediatric disease generally appears to be milder [5,6,7,37]. The presented study reviews the phenotype and clinical course of PSC in a retrospective study at a single paediatric department in Poland.

Primary sclerosing cholangitis is most often diagnosed in the 2nd and 3rd decade of life. The average age of diagnosis in our patients was 13.0. In the studies by Karlsen, Labordy and Fagundes, the average age of the onset of the disease was between 12 and 13, however, all authors report increasingly younger patents, even younger than 5 years of age [1,5,33,38]. Our youngest patient was 7 years old at the onset.

The literature reports that the condition affects men four times more than women [1,4,6,7,22]. Indeed, boys constituted 70% of the patients in our study group. In Denau’s multi-centre study of 781 children with diagnosed PSC girls constituted 40% [4,27]. However Norwegian studies and Weismuller’s examinations indicate a growing number of cases of female patients, especially those with few symptoms or asymptomatic, with a mild course of the disease and with fewer complications. This is also confirmed in Weismuller’s multi-centre study [12].

The clinical presentation of PSC depends on the age of the patient, the advancement of the lesions and underlying diseases. Often the symptoms of PSC such as abdominal pain or loss of body weight correspond with the symptoms of inflammatory bowel disease. Jaundice, which is quite a typical symptom of liver diseases is reported by various authors at 15–25% of the cases of primary sclerosing cholangitis and usually indicates advanced lesions [4,6,7]. As for our patients, abdominal pain was the most common symptom of the clinical presentation while jaundice was reported in only three children (10%). Fagundes in his studies of paediatric patients with diagnosed PSC, concluded that abdominal pain dominated the clinical presentation with jaundice and/or itchy skin being less frequent [5]. Xanthelasmas and disorders of blood clotting most often indicates a significant advancement of hepatic lesions and the presence of complications. 40–50% of patients are asymptomatic, especially in the first phase of the disease and it’s the abnormal parameters of liver functions in IBD patients that prompt further diagnostics. Indeed, close to 60% of our patients did not experience clinical symptoms when diagnosed with PSC. Feldstein in his study showed that the course of PSC in over 30% of children was asymptomatic and it was the abnormal results of the biochemical tests of the blood performed during routine check-up of IBD patients that prompted further diagnostics [6].

The close correlation between PSC and inflammatory bowel diseases is well known [8,9,10]. It is commonly believed that over 2/3 of the patients with sclerosing cholangitis have inflammatory bowel disease. Data available from France and Japan gives a smaller percentage, about 60 and 40 per cent respectively [38,39]. The lower values for Japan are arguably related to a high percentage of patients with IgG4-associated multi-organ disease (IgG4 associated cholangitis). As for our patients, almost all of them, 28/30 (93.3%) were also diagnosed with inflammatory bowel disease (mainly in the form of ulcerative colitis). Deneau et al. concluded the presence of inflammatory bowel disease in 76% of PSC patients (83% of whom had UC and 17% Crohn’s disease) [4,27]. Interestingly, in his study Fagundes concluded the presence of inflammatory bowel diseases in only 24% of patients with primary sclerosing cholangitis [5]. Olson and Schnug reported coexisting ulcerative colitis and primary sclerosing cholangitis in about 5% of patients with IBD [40]. Our observations led to similar conclusions, 28/338 of patients with IBD—8.3%. The risk of developing PSC increases by 0.5–1% with every year of lasting inflammatory bowel disease. According to Tanaka, 3–8% of patients with ulcerative colitis also have primary sclerosing cholangitis [37]. According to Floreani et al. the coexistence of PSC and IBD concerns about 17% of patients, with a higher frequency among paediatric patients [41]. The above discrepancies may be caused by the fact that it was only those with suspected IBD that had a colonoscopy performed as part of the diagnostics. It is worth reiterating that this examination is recommended in all patients with confirmed primary sclerosing cholangitis. The coexistence of Crohn’s disease and PSC is less frequent—at 3% according to the topic literature—it was even lower in our observations, at 1.5%. The literature reports also a milder course of PSC in patients with coexisting Crohn’s disease than with coexisting ulcerative colitis [8,9,10]. Our observations confirm that. The proportion of children suffering from EC (7%) which was not directly reported is intriguing.

Other authors suggest that pancolitis in PSC patients is prevalent and varies from 35% to 95%. In Wiliamson’s studies, in most cases the inflammatory lesions affected the whole of the large intestine. As for our patients, we also reported pancolitis-type lesions [42]. Only 3/30 (10%) had rectal sparing. Both are believed to be characteristic features of PSC-IBD phenotype. In his study group of 74 patients with PSC Ricciutto observed a more frequent occurrence of backwash ileitis, pancolitis and rectal sparing [43].

Comorbidities. One theory discusses the impact of autoimmunological factors on the occurrence of PSC together with others such as genetic implications, the influence of the microbiota of the gastrointestinal tract, as well as the disorders in the synthesis and transport of the bile acids [2,13,15,18,19].

Most often PSC coexists with autoimmune hepatitis. Biochemical and histopathological features of autoimmune hepatitis are present in about 10–39% of patients with PSC. In our patients, “overlap” syndrome was reported in 7/30 (23%). In Denau’s study the syndrome concerned 33% and 7% of other autoimmune diseases [4,27]. The coexistence of PSC and other autoimmune diseases was lower in Valentino’s study, at 5% [31]. One of our patients was diagnosed with autoimmune pancreatitis, confirmed with laboratory and imaging tests (IgG4 -associated autoimmune syndrome). 13.3% of our patients had a family history of autoimmune diseases. The range in Karlsen’s and Williamson’s study is between 15 and 20 per cent [1,42].

Laboratory results. Laboratory tests which indicate cholestasis and damage to the liver are not pathognomonic for the diagnosis of primary sclerosing cholangitis [21,23]. Elevated activity of GTP was reported in all our patients, while there were raised values of aminotransferases in over 70%. Interestingly, all the patients in Fagundes’ study had increased activity of aminotransferases [5]. As Deneau demonstrated, even slight abnormalities in the values of total bilirubin, GTP and APRI when diagnosing PSC may indicate the worse course of the disease. The lowering of the activity of alkaline phosphatase in adults with PSC is a confirmed positive prognostic [4,27]. However, AP is a less reliable marker of liver disease in the paediatric population due to the wide variability in isozymes originating from bone in the growing period. GTP seems to be a better biochemical marker of biliary disease in paediatrics. In all our patients with diagnosed IBD, we routinely perform liver’s testing (i.e., cholestasis parameters: GGTP, bilirubin), which makes it possible to detect changes early, as the clinical course of PSC in most patients is asymptomatic. However, in patients who did not have Inflammatory Bowel Disease, the studies were performed only when clinical signs (itching of the skin, jaundice, hepatomegaly) appeared, and PSC was very advanced clinically.

ANCA.ASCA. The literature mentions a more frequent presence of the ANCA antibodies in patients with ulcerative colitis and coexisting primary sclerosing cholangitis. In our patients, these antibodies were present in only 28.5% (8/28), while ASCA-positive antibodies were observed in 3 patients with Crohn’s disease and one with ulcerative colitis. Stinton in her studies also confirmed the correlation between elevated values of the ANCA antibodies in patients with PSC and the activity of cholestatic enzymes and damage to the liver [44]. We did not find such correspondence in our patients. Fagundes noted increased ANCA antibodies in the serum of only 14.2% of his patients with PSC [5]. Chandrakumar believes that all patients with confirmed inflammatory bowel disease and co-morbid elevated parameters of cholestasis and positive pANCA must be diagnosed and observed towards primary sclerosing cholangitis [45]. Lee et al. in their Australian studies have confirmed the ANCA antibodies in over 80% of patients with PSC but did not demonstrate the correlation with its comorbidity with inflammatory bowel disease and its type [46].

Radiological examinations. Due to technical advances, MRCP is the most useful technique in children with suspected PSC, with high sensitivity and very high specificity. In the presence of typical imaging symptoms, MRCP is sufficient for the diagnosis of PSC, and thus, the risks associated with liver biopsy could be avoided. As ERCP is challenging in children, it is reserved for rare paediatric cases, when biliary intervention is needed. In present study MRCP was performed in all the children, with children below the ages of 7–8 years under sedation. In all cases MRCP images confirmed typical signs of PSC, as multifocal dilatations of intra and extrahepatic bile ducts [34,47,48,49,50]. Strictures with “beading” and string of pearl appearance were also visible. The MRCP performed on our patients revealed lesions in all the cases, confirming the diagnosis of PSC, mostly in the form of multi-segmental strictures and dilatations. According to Fulcher and Vitellas it is MRCP that has 85–88% sensitivity with 92–99% specificity in the diagnostics of primary sclerosing cholangitis [51,52]. However, it must not be forgotten that MRCP may be negative in small duct PSC, as Miloh’s studies confirm. He demonstrated lesions in the MRCP of 70% of patients with PSC [53,54]. Transabdominal US is considered to be the first line imaging modality in children, and in the presented group it was performed in all patients, although only 32% US exams showed various bile duct abnormalities. However, it should be highlighted, that the presence of typical PSC symptoms in US seemed to be prognostic of possible earlier complications, which we did not observe being mentioned before in the literature [1,25,49]. This, most probably indicates a late diagnosis and more advanced lesions.

Liver biopsy. As liver biopsy is not necessary to establish the PSC diagnosis, the number of patients with liver biopsy available fluctuates from 43% in our case to 96% depending on the clinical centre. However, in our study, 54% of biopsies had either PSC or AIH changes. In all their patients with suspected PSC, Miloh et al. confirmed abnormalities in the histopathological examination of the liver, mainly in the form of varying degrees of fibrosis and cirrhosis. “Onion skin” fibrosis, characteristic for PSC was confirmed in only 26.7% [53,54].

Complications. The prognosis of PSC is disadvantageous. The disease is progressive and leads to liver failure and an increased risk of carcinogenesis. Children generally have an earlier stage of the same disease as adult patients and the disease appears milder. General comorbidities (obesity, fatty liver disease, alcoholic hepatitis, smoking, cardiovascular disease and/or diabetes) are much more prevalent in adult patients and may worsen overall outcomes compared to children. One of the most common complications in the course of PSC are critical strictures within the bile ducts, requiring endoscopic (prosthetic) or surgical interventions. They may concern even 20% of the patients (10–25%) [4,32,55,56]. Among our patients, critical strictures were observed in 16.5% (5 patients). Biliary prostheses were used in all the patients, with complications during the procedures of two patients due to perforations within the bile ducts, not requiring a surgical intervention. The available literature reports the frequency of complications following the procedure of ERCP in PSC patients at 20%. However, this concerns all the patients who have undergone the procedure. According to the literature, critical strictures more often affect patients with ulcerative colitis, also among our patients, 80% were those with UC [55,56]. Among our patients with advanced hepatic lesions, there were less advanced changes in the colon- a lower PUCAI/PCDAI score was shown, but this was not a statistically significant difference.

Many children with PSC progress to end-stage liver disease with the consequent need for liver transplantation which is the only life-saving treatment, although it may reccur in the allograft. None of the patients in the study underwent liver transplantation below 18 years of age, compared to 14% in the largest multi-centre study to date.

Cholangiocarcinoma is a rare but serious complication in pediatric PSC. It occurs in 1% of children in the follow-up, compared to 7–9% of adults [57,58,59,60,61]. According to Deneau, the average time of its onset after diagnosis of PSC is about 6 years in the paediatric population [4,27]. In our patients, increased concentration of the Ca 19-9 marker was observed in 2/30 (6.6%), aged 17 and 15.5. One of them required a liver transplant after reaching the age of 18 due to the clinical presentation, the presence of atypical cells in biliary brushing and increasing liver failure. No cancer-indicating abnormalities were observed in the histopathological and imaging tests of the second patient. Patients with critical strictures within the bile ducts, requiring therapeutic interventions are particularly at risk of developing this type of cancer. Both our patients had such medical history and required biliary prostheses. Unfortunately, the prognosis for patients with cholangiocarcinoma in the course of PSC is highly unfavourable and most of them die within 2 years of the diagnosis. The results of liver transplant are also unsatisfactory, with a five-year survival rate at only about 25% [59,60,61].

Treatment. Because the cause of PSC is unknown, the treatment is mainly symptomatic. The available literature does not convincingly report the effectiveness of ursodeoxycholic acid-based medication in the treatment of primary sclerosing cholangitis, especially with particular focus on preventing the advancement of the disease [4,33,34]. The American Association for the Study of Liver Diseases (AASLD) practice guideline for PSC currently discourages the use of UDCA in adults while there are no current recommendations in paediatrics [62]. Data from paediatric PSC case series have shown that treatment with UDCA improves liver biochemistries and cholestatic parameters; however, the treatment did not result in improved outcomes compared with untreated patients. We observed improved GGT levels in follow-up. Improvements in cholestasis and liver cell damage during our 3 years of follow-up may be associated with routine treatment with ursodezoxycholic acid. However, we have not shown a correlation between laboratory test results and the regression of changes in imaging -cholangio MRI studies. Wunsch also observed a statistically significant reduction in the parameters of liver function (AP, GTP, bilirubin and aminotransferases) during therapy with endoscopic retrograde cholangiopancreatography in PSC patients [63]. Currently high hopes are placed on 24-norursodeoxycholic acid, a UDCA homolog which is said to prevent the periductal fibrosis and the proliferation of hepato- and cholangiocytes.

## 5. Conclusions

1. Monitoring liver markers in IBD patients is important since most PSC cases are asymptomatic and elevated liver markers might be the first sign of the disease.
2. Patients diagnosed with PSC before IBD diagnosis are more likely to have a more aggressive course of the disease.

## Figures and Tables

**Table 1:** Location of changes in the alimentary tract of examined patients with IBD using Paris Classification.

Ulcerative	Colitis *N* = 22	Eosinophilic Colitis *N* = 2	Crohn’s	Disease *N* = 4
Paris Classification	Number of Patients	Number of Patients	Paris Classification	Number of Patients
E1 E2 E3 E4 Other S0 S1	0/22 (0%) 3/22 (13.6%) 2/22 (9.1%) 16/22 (72.7%) 1/22 (4.6%) 17/22 (77.3%) 5/22 (22.7%)	1/2 (50%) 1/2 (50%)	A1L1B2 A1L2B3 A1L3B2	1/4 (25%) 1/4 (25%) 2/4 (50%)
Total	22 (100%)	2 (100%)	Total	4 (100%)

**Table 2:** Laboratory examinations in patients with diagnosed PSC.

	At Diagnosis	Follow-Up- 3 Years	*p*
ALT (U/L)	38	23	0.12
Mean activity (Min-Max)	(9–412)	(9–179)	
AST (U/L)	35.5	30	0.18
Mean activity (Min-Max)	(16–285)	(17–254)	
GGT (U/L)	73	37	0.01
Mean activity (Min-Max)	(48–778	(12–235)	
BLB (µmol/L)	8.95	10.8	0.08
Mean concentration (Min–Max)	(4.08–140.7)	(4.8–45.6)	
APRI index	0.33	0.273	0.28
(Min-Max)	(0.112–2.679)	(0.079–2.759)	

Abbreviations: ALT—alanine aminotransferase; AST—aspartate aminotransferase; GGTP—gamma-glutamylo-transferase; BLB—total bilirubin; APRI—AST to Platelet Ratio Index.

**Table 3:** Complication in the course of PSC in examined patients.

COMPLICATIONS	PSC Diagnosed before IBD	IBD Diagnosed before PSC/toGETHER	Total	
	*N* = 6	*N* = 22	*N* = 30	
Portal hypertension	2/6 (33.3%)	0/22 (0.0%)	2/30 (6.6%)	*p* < 0.05
Liver Cirrhosis	2/6 (33.3%)	0/22 (0.0%)	2/30 (6.6%)	
Critical stricture of bile ducts	3/6 (50.0%)	2/22 (9.1%)	5/30 (16.6%)	

## Data Availability

The statistical analysis and database used to support the findings of this study may be released upon application to the Medical University of Silesia, Department of Pediatrics, who can be contacted by the corresponding author.

## Abbreviations

PSC	primary sclerosing cholangitis
IBD	inflammatory bowel disease
UC	ulcerative colitis
CD	Crohn’s diseae
EC	Eosinophilic Colitis
AIH	Autoimmune hepatitis
AST	aspartate aminotransferase
ALT	alkaline aminotransferase
AP	alkaline phosphatase
BLB	total bilirubin
GTP	glutamyltranspeptidase
APRI	AST-to-platelet-ratio Index
ANCA	antineutrophil cytoplasmic antibodies
ANA	antinuclear antibodies
ASCA	anti Saccharomyces cerevisiae anibodies
anti-SMA	anti smooth muscle antibodies
US	ultrasonography examination
MRCP	magnetic resonance cholangiopancreatography
ERCP	endoscopic retrograde cholangiopancreatography
CCA	cholangiocarcinoma
5-ASA	5-aminosalicylic acid
AZA	azathioprine
UDCA	ursodeoxycholic acid
